# {μ-*N*,*N*′-Bis[1-(2-pyridyl)ethylidene]benzene-1,2-diamine}di-μ-chlorido-bis[diaquanickel(II)] dichloride ethanol disolvate

**DOI:** 10.1107/S1600536808041299

**Published:** 2008-12-20

**Authors:** Farba Bouyagui Tamboura, Mohamed Gaye, Abdou Salam Sall, Aliou Hamady Barry, Youssouph Bah

**Affiliations:** a Département de Chimie, Faculté des Sciences et Techniques, Université Cheikh Anta Diop, Dakar, Senegal; b Département de Chimie, Faculté des Sciences, Université de Nouakchott, Nouakchott, Mauritania; c Département de Chimie, Faculté des Sciences, Université de Conakry, Conakry, Guinea

## Abstract

In the title compound, [Ni_2_Cl_2_(C_20_H_18_N_4_)(H_2_O)_4_]Cl_2_·2C_2_H_6_O, the coordination environment of each Ni^2+^ ion is distorted octa­hedral formed by two N atoms from the Schiff base ligand, two O atoms from water mol­ecules and two chloride anions acting as μ_2_ bridges between the metal ions. The coordinated water mol­ecules are linked to the uncoordinated ethanol mol­ecules and chloride anions by O—H⋯O and O—H⋯Cl hydrogen bonds, although the assignment of some of these is tentative. A weak inter­molecular O—H⋯N inter­action within the ligand is also observed.

## Related literature

For related structures, see: Kelly *et al.* (2005 ▶); Garoufis *et al.* (1998 ▶); Li *et al*. (2005 ▶); Deters *et al.* (2005 ▶); Sengottuvelan *et al*. (2008 ▶).
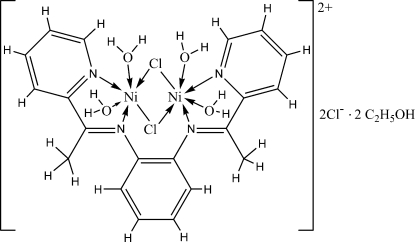


## Experimental

### 

#### Crystal data

[Ni_2_Cl_2_(C_20_H_18_N_4_)(H_2_O)_4_]Cl_2_·2C_2_H_6_O
*M* *_r_* = 737.80
Orthorhombic, 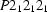

*a* = 13.078 (2) Å
*b* = 13.575 (2) Å
*c* = 17.873 (4) Å
*V* = 3173.06 (10) Å^3^
*Z* = 4
Mo *K*α radiation
μ = 1.57 mm^−1^
*T* = 173 (2) K
0.14 × 0.12 × 0.10 mm

#### Data collection

Nonius KappaCCD diffractometer
Absorption correction: none
13155 measured reflections
8765 independent reflections
6892 reflections with *I* > 2σ(*I*)

#### Refinement

*R*[*F* ^2^ > 2σ(*F* ^2^)] = 0.042
*wR*(*F* ^2^) = 0.096
*S* = 1.04
8765 reflections
385 parameters
8 restraints
H atoms treated by a mixture of independent and constrained refinement
Δρ_max_ = 0.61 e Å^−3^
Δρ_min_ = −0.63 e Å^−3^
Absolute structure: Flack (1983 ▶), 3623 Friedel pairs
Flack parameter: 0.005 (12)

### 

Data collection: *COLLECT* (Nonius, 1998 ▶); cell refinement: *DENZO* (Nonius, 1998 ▶); data reduction: *DENZO*; program(s) used to solve structure: *SHELXS97* (Sheldrick, 2008 ▶); program(s) used to refine structure: *SHELXL97* (Sheldrick, 2008 ▶); molecular graphics: *PLATON* (Spek, 2003 ▶); software used to prepare material for publication: *SHELXL97*.

## Supplementary Material

Crystal structure: contains datablocks I, global. DOI: 10.1107/S1600536808041299/hb2846sup1.cif

Structure factors: contains datablocks I. DOI: 10.1107/S1600536808041299/hb2846Isup2.hkl

Additional supplementary materials:  crystallographic information; 3D view; checkCIF report

## Figures and Tables

**Table 1:** Selected bond lengths (Å)

Ni1—N1	2.071 (3)
Ni1—N2	2.073 (3)
Ni1—O1	2.072 (2)
Ni1—O2	2.085 (2)
Ni1—Cl2	2.3985 (8)
Ni1—Cl1	2.4323 (8)
Ni2—N4	2.063 (2)
Ni2—N3	2.077 (3)
Ni2—O3	2.087 (2)
Ni2—O4	2.097 (2)
Ni2—Cl2	2.3778 (8)
Ni2—Cl1	2.4331 (8)

**Table 2:** Hydrogen-bond geometry (Å, °)

*D*—H⋯*A*	*D*—H	H⋯*A*	*D*⋯*A*	*D*—H⋯*A*
O1—H*W*1*A*⋯O5^i^	0.888 (19)	1.98 (3)	2.781 (3)	150 (4)
O1—H*W*1*B*⋯Cl3^i^	0.934 (18)	2.175 (19)	3.107 (2)	176 (4)
O2—H*W*2*A*⋯Cl3^i^	0.916 (19)	2.21 (2)	3.104 (3)	165 (4)
O2—H*W*2*B*⋯Cl4	0.942 (19)	2.122 (19)	3.064 (3)	179 (4)
O3—H*W*3*A*⋯O5^i^	0.939 (19)	1.87 (2)	2.787 (3)	163 (4)
O3—H*W*3*B*⋯Cl3^ii^	0.919 (18)	2.215 (19)	3.133 (2)	176 (4)
O4—H*W*4*A*⋯O6^ii^	0.922 (19)	1.79 (2)	2.702 (4)	168 (4)
O4—H*W*4*B*⋯Cl3^ii^	0.921 (19)	2.27 (2)	3.136 (3)	157 (4)
O5—H1*A*⋯O1^iii^	0.84	1.96	2.781 (3)	166
O6—H2*A*⋯O4^iv^	0.84	2.18	2.702 (4)	121
O6—H2*A*⋯N4^iv^	0.84	2.66	3.467 (4)	163

Symmetry codes: (i) 
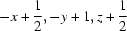
; (ii) 
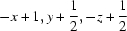
; (iii) 
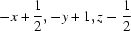
; (iv) 
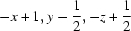
.

**Fig. 1.**
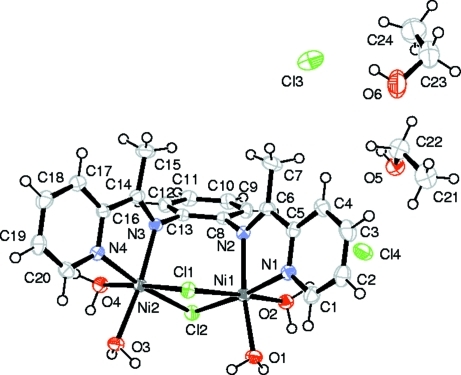
The molecular structure of (I) with displacement ellipsoids for the nno-hydrogen atoms plotted at the 50% probability level.

**Fig. 2.**
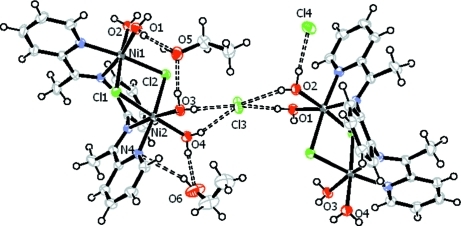
The packing for (I): the broken lines stand for hydrogen bonds.

## supplementary crystallographic information

### Comment

The structure of the title compound, (I), is shown in Fig. 1. In the complex, the ligand coordinates the two metal ions via the two imine N atoms and the two pyridyl nitrogen atoms (one of each type per metal ion). Each metal ion is coordinated by two oxygen atoms from water molecules and two chloride anions (Table 1). Thus, the metal ions are in facial N_2_O_2_Cl_2_ coordination environments and are connected by two chloride anions bridges to yield a binuclear complex. The Ni···Ni distance spanned by the two chloride ion bridges is 3.9615 (16) Å.  The Ni—Cl distances in (I) are longer than distances observed in other Ni^II^ coordination complexes (Li *et al.*, 2005; Deters *et al.*, 2005; Sengottuvelan *et al.*, 2008). Two chloride ions are present in the asymmetric unit to compensate for the doubly positive charge of the complex and hydrogen bonds (Table 2) help to establish the packing.

### Experimental

To a mixture of 1 g (9.2 mmol) 1,2-diaminobenzene and 100 ml of ethanol was added dropwise a solution of 2.24 g (18.46 mmol) of 2-acetylpyridine. The resulting mixture was stirred under reflux for 60 min. A solution of 2.19 g (9.2 mmol) of NiCl_2_.6H_2_O was added. After cooling the solution was filtered and the solvent evaporated to dryness. The resulting yellow solid was recrystallized by diffusion of toluene in the ethanolic solution of the compound and green prisms of (I) were obtained (5.2 g; 75.00°) after one week. IR (cm^-1^,KBr): 3372, 1595, 1570. Analysis calculated for C_24_H_38_Cl_4_N_4_O_6_Ni_2_: C 39.07, H 5.19, N 7.59 °; found: C 39.01, H 5.21, N 7.60 °.

### Refinement

The water H atoms were located from difference maps and refined with distance restraints of 0.96±0.02Å.  The other H atoms were placed geometrically (C—H = 0.95–0.99Å, O—H = 0.84Å) and refined as riding with *U*_iso_(H) = 1.2*U*_eq_(C) or 1.5*U*_eq_(methyl C, O).  The refinement scheme has led to some very short intermolecular H···H contacts and the location of the water H atoms should be regarded as less certain.

### Crystal data

**Table:**

[Ni_2_Cl_2_(C_20_H_18_N_4_)(H_2_O)_4_]Cl_2_·2C_2_H_6_O	*F*(000) = 1528
*M**_r_* = 737.80	*D*_x_ = 1.544 Mg m^−^^3^
Orthorhombic, *P*2_1_2_1_2_1_	Mo *K*α radiation, λ = 0.71073 Å
Hall symbol: P 2ac 2ab	Cell parameters from 5114 reflections
*a* = 13.078 (2) Å	θ = 1.0–30.0°
*b* = 13.575 (2) Å	µ = 1.57 mm^−^^1^
*c* = 17.873 (4) Å	*T* = 173 K
*V* = 3173.06 (10) Å^3^	Prism, green
*Z* = 4	0.14 × 0.12 × 0.10 mm

### Data collection

**Table:**

KappaCCD diffractometer	6892 reflections with *I* > 2σ(*I*)
Radiation source: fine-focus sealed tube	*R*_int_ = 0.0000
graphite	θ_max_ = 30.0°, θ_min_ = 3.2°
π and ω scans	*h* = −18→18
13155 measured reflections	*k* = −19→19
8765 independent reflections	*l* = −25→25

### Refinement

**Table:**

Refinement on *F*^2^	Secondary atom site location: difference Fourier map
Least-squares matrix: full	Hydrogen site location: inferred from neighbouring sites
*R*[*F*^2^ > 2σ(*F*^2^)] = 0.042	H atoms treated by a mixture of independent and constrained refinement
*wR*(*F*^2^) = 0.096	*w* = 1/[σ^2^(*F*_o_^2^) + (0.0393*P*)^2^ + 1.0903*P*] where *P* = (*F*_o_^2^ + 2*F*_c_^2^)/3
*S* = 1.04	(Δ/σ)_max_ = 0.006
8765 reflections	Δρ_max_ = 0.61 e Å^−^^3^
385 parameters	Δρ_min_ = −0.62 e Å^−^^3^
8 restraints	Absolute structure: Flack (1983), 3623 Friedel pairs
Primary atom site location: structure-invariant direct methods	Flack parameter: 0.005 (12)

### Special details

**Table:**

Geometry. All e.s.d.'s (except the e.s.d. in the dihedral angle between two l.s. planes) are estimated using the full covariance matrix. The cell e.s.d.'s are taken into account individually in the estimation of e.s.d.'s in distances, angles and torsion angles; correlations between e.s.d.'s in cell parameters are only used when they are defined by crystal symmetry. An approximate (isotropic) treatment of cell e.s.d.'s is used for estimating e.s.d.'s involving l.s. planes.
Refinement. Refinement on *F*^2^ for ALL reflections except for 0 with very negative *F*^2^ or flagged by the user for potential systematic errors. Weighted *R*-factors *wR* and all goodnesses of fit *S* are based on *F*^2^, conventional *R*-factors *R* are based on *F*, with *F* set to zero for negative *F*^2^. The observed criterion of *F*^2^ > σ(*F*^2^) is used only for calculating _R_factor_obs *etc*. and is not relevant to the choice of reflections for refinement. *R*-factors based on *F*^2^ are statistically about twice as large as those based on *F*, and *R*- factors based on ALL data will be even larger.

### Fractional atomic coordinates and isotropic or equivalent isotropic displacement parameters (Å^2^)

**Table:**

	*x*	*y*	*z*	*U*_iso_*/*U*_eq_	
Ni1	0.35695 (3)	0.50399 (3)	0.39085 (2)	0.01943 (9)	
Ni2	0.61577 (3)	0.50829 (3)	0.40432 (2)	0.01891 (9)	
Cl1	0.48398 (6)	0.39219 (5)	0.44251 (5)	0.02207 (16)	
Cl2	0.48535 (6)	0.62991 (5)	0.39662 (5)	0.02335 (17)	
Cl3	0.28973 (9)	0.25896 (7)	−0.01232 (6)	0.0461 (3)	
Cl4	0.08115 (8)	0.53903 (7)	0.24677 (6)	0.0400 (2)	
O1	0.30614 (18)	0.53214 (16)	0.49855 (13)	0.0255 (5)	
HW1A	0.351 (3)	0.519 (3)	0.5344 (18)	0.050*	
HW1B	0.279 (3)	0.5959 (17)	0.497 (2)	0.050*	
O2	0.25390 (18)	0.60814 (18)	0.35001 (14)	0.0256 (6)	
HW2A	0.228 (3)	0.645 (3)	0.3889 (18)	0.050*	
HW2B	0.200 (2)	0.587 (3)	0.319 (2)	0.050*	
O3	0.64419 (18)	0.53720 (16)	0.51712 (13)	0.0245 (5)	
HW3A	0.588 (2)	0.535 (3)	0.549 (2)	0.050*	
HW3B	0.660 (3)	0.6031 (15)	0.517 (2)	0.050*	
O4	0.72880 (18)	0.61205 (17)	0.37763 (14)	0.0253 (5)	
HW4A	0.7962 (17)	0.594 (3)	0.370 (2)	0.050*	
HW4B	0.743 (3)	0.651 (3)	0.4184 (17)	0.050*	
O5	0.03498 (19)	0.43711 (17)	0.09927 (15)	0.0345 (6)	
H1A	0.0755	0.4509	0.0644	0.050*	
O6	0.0747 (2)	0.0506 (3)	0.1231 (2)	0.0660 (10)	
H2A	0.1320	0.0246	0.1155	0.050*	
N1	0.2552 (2)	0.3885 (2)	0.37723 (17)	0.0229 (6)	
N2	0.38534 (19)	0.46316 (17)	0.28105 (15)	0.0190 (5)	
N3	0.60457 (19)	0.46709 (18)	0.29271 (15)	0.0193 (6)	
N4	0.71873 (19)	0.39336 (18)	0.39947 (16)	0.0197 (5)	
C1	0.1904 (3)	0.3521 (3)	0.4284 (2)	0.0299 (8)	
H1	0.1873	0.3825	0.4762	0.036*	
C2	0.1276 (3)	0.2716 (3)	0.4140 (2)	0.0355 (9)	
H2	0.0824	0.2476	0.4514	0.043*	
C3	0.1321 (3)	0.2272 (3)	0.3446 (2)	0.0348 (9)	
H3	0.0899	0.1722	0.3334	0.042*	
C4	0.1993 (3)	0.2642 (2)	0.2912 (2)	0.0282 (8)	
H4	0.2034	0.2350	0.2430	0.034*	
C5	0.2603 (2)	0.3446 (2)	0.3093 (2)	0.0227 (7)	
C6	0.3333 (2)	0.3895 (2)	0.25517 (19)	0.0246 (7)	
C7	0.3406 (3)	0.3484 (3)	0.1785 (2)	0.0370 (9)	
H7A	0.2776	0.3631	0.1510	0.056*	
H7B	0.3499	0.2768	0.1814	0.056*	
H7C	0.3990	0.3779	0.1526	0.056*	
C8	0.4461 (2)	0.5215 (2)	0.23195 (18)	0.0206 (7)	
C9	0.3971 (3)	0.5831 (2)	0.1807 (2)	0.0271 (8)	
H9	0.3250	0.5799	0.1751	0.032*	
C10	0.4531 (3)	0.6488 (2)	0.1380 (2)	0.0291 (8)	
H10	0.4192	0.6919	0.1043	0.035*	
C11	0.5586 (3)	0.6515 (3)	0.1445 (2)	0.0296 (8)	
H11	0.5972	0.6962	0.1149	0.036*	
C12	0.6079 (3)	0.5888 (2)	0.1942 (2)	0.0274 (8)	
H12	0.6804	0.5899	0.1981	0.033*	
C13	0.5517 (2)	0.5246 (2)	0.23829 (18)	0.0205 (7)	
C14	0.6560 (2)	0.3905 (2)	0.27341 (19)	0.0237 (7)	
C15	0.6549 (3)	0.3448 (3)	0.1975 (2)	0.0386 (10)	
H15A	0.6486	0.2731	0.2023	0.058*	
H15B	0.7187	0.3607	0.1713	0.058*	
H15C	0.5968	0.3705	0.1689	0.058*	
C16	0.7215 (2)	0.3468 (2)	0.33302 (19)	0.0211 (7)	
C17	0.7835 (3)	0.2657 (2)	0.3212 (2)	0.0266 (8)	
H17	0.7858	0.2345	0.2736	0.032*	
C18	0.8422 (3)	0.2308 (2)	0.3800 (2)	0.0314 (8)	
H18	0.8847	0.1748	0.3734	0.038*	
C19	0.8382 (3)	0.2780 (3)	0.4480 (2)	0.0324 (8)	
H19	0.8780	0.2550	0.4889	0.039*	
C20	0.7753 (3)	0.3597 (2)	0.4559 (2)	0.0267 (8)	
H20	0.7725	0.3924	0.5028	0.032*	
C21	−0.0361 (4)	0.3042 (3)	0.1739 (3)	0.0508 (12)	
H21A	−0.0340	0.2328	0.1817	0.076*	
H21B	−0.1056	0.3241	0.1603	0.076*	
H21C	−0.0158	0.3378	0.2201	0.076*	
C22	0.0361 (3)	0.3317 (3)	0.1123 (2)	0.0417 (10)	
H22A	0.1062	0.3107	0.1258	0.050*	
H22B	0.0163	0.2969	0.0658	0.050*	
C23	0.0117 (4)	0.0366 (4)	0.0593 (3)	0.0704 (15)	
H23A	−0.0561	0.0662	0.0694	0.084*	
H23B	0.0015	−0.0350	0.0518	0.084*	
C24	0.0527 (4)	0.0792 (4)	−0.0104 (3)	0.0676 (15)	
H24A	0.0395	0.1503	−0.0111	0.101*	
H24B	0.0193	0.0482	−0.0534	0.101*	
H24C	0.1266	0.0675	−0.0129	0.101*	

### Atomic displacement parameters (Å^2^)

**Table:**

	*U*^11^	*U*^22^	*U*^33^	*U*^12^	*U*^13^	*U*^23^
Ni1	0.02046 (19)	0.01869 (19)	0.0192 (2)	−0.00053 (16)	0.00040 (15)	−0.00077 (17)
Ni2	0.02056 (19)	0.01757 (18)	0.0186 (2)	0.00017 (16)	−0.00013 (15)	−0.00029 (17)
Cl1	0.0228 (4)	0.0177 (3)	0.0257 (4)	−0.0007 (3)	0.0004 (3)	0.0026 (3)
Cl2	0.0246 (4)	0.0183 (3)	0.0272 (4)	0.0005 (3)	−0.0005 (4)	−0.0005 (3)
Cl3	0.0681 (7)	0.0345 (5)	0.0358 (6)	0.0194 (5)	0.0085 (5)	0.0105 (4)
Cl4	0.0453 (6)	0.0444 (5)	0.0303 (5)	−0.0074 (4)	−0.0043 (4)	−0.0064 (4)
O1	0.0302 (13)	0.0273 (12)	0.0188 (13)	0.0035 (10)	−0.0009 (10)	−0.0024 (10)
O2	0.0262 (14)	0.0255 (12)	0.0252 (15)	0.0027 (10)	−0.0005 (11)	−0.0031 (10)
O3	0.0289 (13)	0.0241 (11)	0.0206 (13)	−0.0015 (10)	0.0005 (10)	−0.0034 (9)
O4	0.0261 (12)	0.0250 (12)	0.0250 (14)	−0.0031 (10)	0.0020 (11)	0.0021 (10)
O5	0.0393 (15)	0.0381 (13)	0.0259 (14)	−0.0034 (11)	0.0030 (12)	0.0069 (11)
O6	0.0325 (16)	0.109 (3)	0.056 (2)	−0.0077 (17)	−0.0038 (16)	0.001 (2)
N1	0.0230 (15)	0.0211 (13)	0.0246 (16)	0.0015 (11)	−0.0007 (12)	0.0003 (12)
N2	0.0198 (13)	0.0165 (12)	0.0208 (14)	0.0000 (10)	0.0017 (11)	0.0008 (10)
N3	0.0207 (13)	0.0199 (13)	0.0172 (14)	−0.0013 (10)	0.0008 (11)	−0.0003 (10)
N4	0.0204 (13)	0.0178 (12)	0.0209 (15)	0.0001 (10)	0.0009 (12)	0.0026 (11)
C1	0.0251 (18)	0.0332 (18)	0.032 (2)	−0.0022 (15)	0.0035 (16)	0.0010 (16)
C2	0.026 (2)	0.0381 (19)	0.042 (2)	−0.0064 (16)	0.0056 (17)	0.0087 (17)
C3	0.031 (2)	0.0239 (17)	0.049 (3)	−0.0091 (16)	−0.0048 (19)	0.0004 (16)
C4	0.0275 (18)	0.0246 (17)	0.032 (2)	−0.0016 (15)	−0.0048 (16)	−0.0004 (15)
C5	0.0212 (17)	0.0207 (16)	0.0264 (19)	0.0012 (13)	0.0000 (14)	0.0026 (14)
C6	0.0275 (18)	0.0236 (16)	0.0226 (18)	0.0038 (14)	−0.0018 (14)	−0.0021 (14)
C7	0.047 (2)	0.037 (2)	0.027 (2)	−0.0130 (18)	0.0027 (18)	−0.0100 (16)
C8	0.0257 (16)	0.0181 (15)	0.0180 (16)	−0.0008 (12)	0.0016 (13)	0.0004 (12)
C9	0.0247 (18)	0.0272 (17)	0.029 (2)	0.0040 (14)	−0.0031 (15)	0.0005 (14)
C10	0.038 (2)	0.0267 (17)	0.0228 (19)	0.0036 (15)	−0.0013 (16)	0.0075 (14)
C11	0.038 (2)	0.0268 (17)	0.024 (2)	−0.0041 (15)	0.0027 (16)	0.0071 (14)
C12	0.0257 (18)	0.0294 (17)	0.0270 (19)	−0.0030 (14)	0.0017 (15)	0.0016 (14)
C13	0.0257 (16)	0.0183 (15)	0.0176 (16)	−0.0007 (12)	0.0001 (13)	−0.0016 (12)
C14	0.0236 (17)	0.0240 (16)	0.0235 (18)	−0.0021 (14)	0.0036 (14)	−0.0023 (13)
C15	0.046 (2)	0.044 (2)	0.025 (2)	0.0153 (19)	−0.0069 (18)	−0.0112 (17)
C16	0.0203 (16)	0.0211 (15)	0.0220 (18)	−0.0013 (13)	0.0003 (14)	0.0006 (13)
C17	0.0283 (19)	0.0249 (17)	0.026 (2)	0.0004 (14)	0.0017 (15)	−0.0005 (14)
C18	0.0305 (19)	0.0247 (16)	0.039 (2)	0.0076 (14)	0.0005 (17)	0.0000 (16)
C19	0.0262 (19)	0.0327 (19)	0.038 (2)	0.0077 (15)	−0.0058 (17)	0.0054 (16)
C20	0.0284 (18)	0.0288 (17)	0.0230 (19)	0.0004 (14)	−0.0031 (15)	0.0018 (14)
C21	0.053 (3)	0.049 (2)	0.050 (3)	−0.010 (2)	−0.002 (2)	0.015 (2)
C22	0.044 (2)	0.038 (2)	0.044 (3)	0.0019 (17)	0.000 (2)	0.0052 (18)
C23	0.042 (3)	0.099 (4)	0.070 (4)	−0.009 (3)	−0.003 (3)	−0.013 (3)
C24	0.065 (3)	0.096 (4)	0.042 (3)	−0.011 (3)	0.007 (3)	−0.001 (3)

### Geometric parameters (Å, °)

**Table:**

Ni1—N1	2.071 (3)	C5—C6	1.490 (5)
Ni1—N2	2.073 (3)	C6—C7	1.482 (5)
Ni1—O1	2.072 (2)	C7—H7A	0.9800
Ni1—O2	2.085 (2)	C7—H7B	0.9800
Ni1—Cl2	2.3985 (8)	C7—H7C	0.9800
Ni1—Cl1	2.4323 (8)	C8—C13	1.386 (4)
Ni2—N4	2.063 (2)	C8—C9	1.396 (4)
Ni2—N3	2.077 (3)	C9—C10	1.384 (5)
Ni2—O3	2.087 (2)	C9—H9	0.9500
Ni2—O4	2.097 (2)	C10—C11	1.385 (5)
Ni2—Cl2	2.3778 (8)	C10—H10	0.9500
Ni2—Cl1	2.4331 (8)	C11—C12	1.390 (5)
O1—HW1A	0.888 (19)	C11—H11	0.9500
O1—HW1B	0.934 (18)	C12—C13	1.386 (4)
O2—HW2A	0.916 (19)	C12—H12	0.9500
O2—HW2B	0.942 (19)	C14—C16	1.490 (5)
O3—HW3A	0.939 (19)	C14—C15	1.493 (5)
O3—HW3B	0.919 (18)	C15—H15A	0.9800
O4—HW4A	0.922 (19)	C15—H15B	0.9800
O4—HW4B	0.921 (19)	C15—H15C	0.9800
O5—C22	1.450 (4)	C16—C17	1.384 (5)
O5—H1A	0.8400	C17—C18	1.385 (5)
O6—C23	1.418 (6)	C17—H17	0.9500
O6—H2A	0.8400	C18—C19	1.374 (5)
N1—C1	1.340 (4)	C18—H18	0.9500
N1—C5	1.354 (5)	C19—C20	1.389 (5)
N2—C6	1.295 (4)	C19—H19	0.9500
N2—C8	1.424 (4)	C20—H20	0.9500
N3—C14	1.285 (4)	C21—C22	1.498 (6)
N3—C13	1.426 (4)	C21—H21A	0.9800
N4—C20	1.332 (4)	C21—H21B	0.9800
N4—C16	1.346 (4)	C21—H21C	0.9800
C1—C2	1.391 (5)	C22—H22A	0.9900
C1—H1	0.9500	C22—H22B	0.9900
C2—C3	1.381 (6)	C23—C24	1.475 (7)
C2—H2	0.9500	C23—H23A	0.9900
C3—C4	1.390 (5)	C23—H23B	0.9900
C3—H3	0.9500	C24—H24A	0.9800
C4—C5	1.390 (5)	C24—H24B	0.9800
C4—H4	0.9500	C24—H24C	0.9800
			
N1—Ni1—O1	92.45 (11)	N2—C6—C5	114.9 (3)
N1—Ni1—N2	78.55 (11)	C7—C6—C5	119.2 (3)
O1—Ni1—N2	170.54 (10)	C6—C7—H7A	109.5
N1—Ni1—O2	93.27 (10)	C6—C7—H7B	109.5
O1—Ni1—O2	89.59 (9)	H7A—C7—H7B	109.5
N2—Ni1—O2	88.03 (10)	C6—C7—H7C	109.5
N1—Ni1—Cl2	174.04 (8)	H7A—C7—H7C	109.5
O1—Ni1—Cl2	93.04 (7)	H7B—C7—H7C	109.5
N2—Ni1—Cl2	96.08 (7)	C13—C8—C9	119.5 (3)
O2—Ni1—Cl2	89.10 (7)	C13—C8—N2	121.5 (3)
N1—Ni1—Cl1	90.64 (8)	C9—C8—N2	118.8 (3)
O1—Ni1—Cl1	88.94 (7)	C10—C9—C8	120.4 (3)
N2—Ni1—Cl1	94.02 (7)	C10—C9—H9	119.8
O2—Ni1—Cl1	175.89 (7)	C8—C9—H9	119.8
Cl2—Ni1—Cl1	87.14 (3)	C9—C10—C11	119.8 (3)
N4—Ni2—N3	78.58 (11)	C9—C10—H10	120.1
N4—Ni2—O3	93.82 (10)	C11—C10—H10	120.1
N3—Ni2—O3	172.26 (10)	C10—C11—C12	120.0 (3)
N4—Ni2—O4	92.20 (9)	C10—C11—H11	120.0
N3—Ni2—O4	90.69 (10)	C12—C11—H11	120.0
O3—Ni2—O4	88.16 (9)	C13—C12—C11	120.2 (3)
N4—Ni2—Cl2	172.31 (8)	C13—C12—H12	119.9
N3—Ni2—Cl2	94.64 (7)	C11—C12—H12	119.9
O3—Ni2—Cl2	93.04 (7)	C12—C13—C8	120.1 (3)
O4—Ni2—Cl2	91.50 (7)	C12—C13—N3	118.3 (3)
N4—Ni2—Cl1	89.10 (7)	C8—C13—N3	121.5 (3)
N3—Ni2—Cl1	92.58 (7)	N3—C14—C16	115.5 (3)
O3—Ni2—Cl1	88.68 (7)	N3—C14—C15	125.1 (3)
O4—Ni2—Cl1	176.66 (8)	C16—C14—C15	119.3 (3)
Cl2—Ni2—Cl1	87.59 (3)	C14—C15—H15A	109.5
Ni1—Cl1—Ni2	88.46 (3)	C14—C15—H15B	109.5
Ni2—Cl2—Ni1	90.56 (3)	H15A—C15—H15B	109.5
Ni1—O1—HW1A	115 (3)	C14—C15—H15C	109.5
Ni1—O1—HW1B	106 (3)	H15A—C15—H15C	109.5
HW1A—O1—HW1B	116 (4)	H15B—C15—H15C	109.5
Ni1—O2—HW2A	110 (3)	N4—C16—C17	121.6 (3)
Ni1—O2—HW2B	119 (2)	N4—C16—C14	115.4 (3)
HW2A—O2—HW2B	110 (4)	C17—C16—C14	123.0 (3)
Ni2—O3—HW3A	117 (3)	C16—C17—C18	118.7 (3)
Ni2—O3—HW3B	102 (3)	C16—C17—H17	120.7
HW3A—O3—HW3B	103 (3)	C18—C17—H17	120.7
Ni2—O4—HW4A	122 (3)	C19—C18—C17	119.5 (3)
Ni2—O4—HW4B	110 (3)	C19—C18—H18	120.3
HW4A—O4—HW4B	94 (3)	C17—C18—H18	120.3
C22—O5—H1A	109.5	C18—C19—C20	119.0 (3)
C23—O6—H2A	109.5	C18—C19—H19	120.5
C1—N1—C5	118.8 (3)	C20—C19—H19	120.5
C1—N1—Ni1	127.2 (2)	N4—C20—C19	121.7 (3)
C5—N1—Ni1	114.0 (2)	N4—C20—H20	119.1
C6—N2—C8	120.2 (3)	C19—C20—H20	119.1
C6—N2—Ni1	116.8 (2)	C22—C21—H21A	109.5
C8—N2—Ni1	122.33 (19)	C22—C21—H21B	109.5
C14—N3—C13	120.9 (3)	H21A—C21—H21B	109.5
C14—N3—Ni2	116.0 (2)	C22—C21—H21C	109.5
C13—N3—Ni2	122.83 (19)	H21A—C21—H21C	109.5
C20—N4—C16	119.5 (3)	H21B—C21—H21C	109.5
C20—N4—Ni2	126.2 (2)	O5—C22—C21	111.0 (3)
C16—N4—Ni2	114.2 (2)	O5—C22—H22A	109.4
N1—C1—C2	122.5 (3)	C21—C22—H22A	109.4
N1—C1—H1	118.8	O5—C22—H22B	109.4
C2—C1—H1	118.8	C21—C22—H22B	109.4
C3—C2—C1	118.9 (3)	H22A—C22—H22B	108.0
C3—C2—H2	120.6	O6—C23—C24	114.5 (4)
C1—C2—H2	120.6	O6—C23—H23A	108.6
C2—C3—C4	119.1 (3)	C24—C23—H23A	108.6
C2—C3—H3	120.5	O6—C23—H23B	108.6
C4—C3—H3	120.5	C24—C23—H23B	108.6
C5—C4—C3	119.1 (3)	H23A—C23—H23B	107.6
C5—C4—H4	120.5	C23—C24—H24A	109.5
C3—C4—H4	120.5	C23—C24—H24B	109.5
N1—C5—C4	121.7 (3)	H24A—C24—H24B	109.5
N1—C5—C6	115.8 (3)	C23—C24—H24C	109.5
C4—C5—C6	122.5 (3)	H24A—C24—H24C	109.5
N2—C6—C7	126.0 (3)	H24B—C24—H24C	109.5

### Hydrogen-bond geometry (Å, °)

**Table:**

*D*—H···*A*	*D*—H	H···*A*	*D*···*A*	*D*—H···*A*
O1—HW1A···O5^i^	0.89 (2)	1.98 (3)	2.781 (3)	150 (4)
O1—HW1B···Cl3^i^	0.93 (2)	2.17 (2)	3.107 (2)	176 (4)
O2—HW2A···Cl3^i^	0.92 (2)	2.21 (2)	3.104 (3)	165 (4)
O2—HW2B···Cl4	0.94 (2)	2.12 (2)	3.064 (3)	179 (4)
O3—HW3A···O5^i^	0.94 (2)	1.87 (2)	2.787 (3)	163 (4)
O3—HW3B···Cl3^ii^	0.92 (2)	2.22 (2)	3.133 (2)	176 (4)
O4—HW4A···O6^ii^	0.92 (2)	1.79 (2)	2.702 (4)	168 (4)
O4—HW4B···Cl3^ii^	0.92 (2)	2.27 (2)	3.136 (3)	157 (4)
O5—H1A···O1^iii^	0.84	1.96	2.781 (3)	166
O6—H2A···O4^iv^	0.84	2.18	2.702 (4)	121
O6—H2A···N4^iv^	0.84	2.66	3.467 (4)	163

Symmetry codes: (i) −*x*+1/2, −*y*+1, *z*+1/2; (ii) −*x*+1, *y*+1/2, −*z*+1/2; (iii) −*x*+1/2, −*y*+1, *z*−1/2; (iv) −*x*+1, *y*−1/2, −*z*+1/2.
